# Combination of Paclitaxel and MG1 oncolytic virus as a successful strategy for breast cancer treatment

**DOI:** 10.1186/s13058-016-0744-y

**Published:** 2016-08-08

**Authors:** Marie-Claude Bourgeois-Daigneault, Lauren Elizabeth St-Germain, Dominic Guy Roy, Adrian Pelin, Amelia Sadie Aitken, Rozanne Arulanandam, Theresa Falls, Vanessa Garcia, Jean-Simon Diallo, John Cameron Bell

**Affiliations:** 1 Ottawa Hospital Research Institute, Centre for Innovative Cancer Research, Ottawa, K1H 8L6 Canada; 2 Department of Biochemistry, Microbiology and Immunology, University of Ottawa, Ottawa, K1H 8M5 Canada

**Keywords:** Breast Cancer, Triple-negative breast cancer, TNBC, Oncolytic virus, Rhabdovirus Maraba-MG1, Paclitaxel, Viral Sensitizer, Combination therapy

## Abstract

**Background:**

Breast cancer is the most common malignant disease amongst Western women. The lack of treatment options for patients with chemotherapy-resistant or recurrent cancers is pushing the field toward the rapid development of novel therapies. The use of oncolytic viruses is a promising approach for the treatment of disseminated diseases like breast cancer, with the first candidate recently approved by the Food and Drug Administration for use in patients. In this report, we demonstrate the compatibility of oncolytic virotherapy and chemotherapy using various murine breast cancer models. This one-two punch has been explored in the past by several groups with different viruses and drugs and was shown to be a successful approach. Our strategy is to combine Paclitaxel, one of the most common drugs used to treat patients with breast cancer, and the oncolytic Rhabdovirus Maraba-MG1, a clinical trial candidate in a study currently recruiting patients with late-stage metastatic cancer.

**Methods:**

We used the EMT6, 4 T1 and E0771 murine breast cancer models to evaluate in vitro and in vivo the effects of co-treatment with MG1 and Paclitaxel. Treatment-induced cytotoxicity was assessed and plaque assays, flow cytometry, microscopy and immunocytochemistry analysis were performed to quantify virus production and transgene expression. Orthotopically implanted tumors were measured during and after treatment to evaluate efficacy and Kaplan-Meier survival curves were generated.

**Results:**

Our data demonstrate not only the compatibility of the treatments, but also their synergistic cytopathic activity. With Paclitaxel, EMT6 and 4 T1 tumors demonstrated increased virus production both in vitro and in vivo. Our results also show that Paclitaxel does not impair the safety profile of the virus treatment. Importantly, when combined, MG1 and the drug controlled tumor growth and prolonged survival.

**Conclusions:**

The combination of MG1 and Paclitaxel improved efficacy in all of the breast cancer models we tested and thus is a promising alternative approach for the treatment of patients with refractory breast cancer. Our strategy has potential for rapid translation to the clinic, given the current clinical status of both agents.

**Electronic supplementary material:**

The online version of this article (doi:10.1186/s13058-016-0744-y) contains supplementary material, which is available to authorized users.

## Background

Breast cancer is a highly aggressive disease with most of the deaths resulting from metastases within the first three years upon diagnosis [1]. Metastatic human triple-negative breast cancer (TNBC) has the worst prognosis among all types of breast cancer, with high risk of rapid recurrence and shortened survival [2]. TNBC is deficient in the expression of estrogen receptor, progesterone receptor and human epidermal growth factor receptor 2, and thus, is refractory to conventional breast cancer hormonal therapy such as Tamoxifen. The main therapeutic option with surgery is chemotherapy, but some subsets of tumor are resistant and the prognosis for these patients is poor [3]. The standard of care for TNBC is the administration of anthracyclines and/or taxanes [2]. Paclitaxel (PAC), also called Taxol, is a cancer chemotherapeutic agent of the taxane family that acts by stabilizing microtubules and thus preventing cell division [4]. PAC is commonly used as monotherapy or in combination with different agents. Significant effort is currently being directed toward improving its efficacy and developing alternate strategies for the treatment of chemotherapy-resistant and recurrent disease.

A novel strategy being explored for the treatment of metastatic diseases such as TNBC is the use of oncolytic viruses (OV). Several candidates are currently undergoing clinical trials and are considered promising approaches for the treatment of various cancers including TNBC [5]. At the forefront of this field is T-Vec, a herpes virus that was successfully tested in a phase III study in melanoma and was approved in 2015 by the Food and Drug Administration for clinical use. OVs specifically replicate in and destroy tumor cells by several mechanisms including direct oncolysis [6]. The rhabdovirus family members, vesicular stomatitis virus (VSV) and maraba, were first identified as oncolytic agents by our group [7, 8]. The tumor specificity of these viruses is conferred by the capacity of normal cells, but not tumor cells, to respond to antiviral interferons (IFN) [7, 8]. Variants with a greater therapeutic index, VSVΔ51 and Maraba MG1, were subsequently developed for clinical use [8, 9]. Importantly, enrolling recently began for a clinical trial using MG1 both as a stand-alone therapy and in a vaccination strategy in patients with late-stage disseminated disease (NCT02285816).

A means to further improve the efficacy of the virus is to augment its replication in the tumor. In a previous study, we identified drugs, so-called virus sensitizers (VSe), that enhanced VSV replication in a tumor-specific manner [10]. The compound identified as VSe12 in that study is PAC and it demonstrated the ability to substantially increase viral replication in vitro. Another VSe, colchicine, affects microtubule dynamics and was also the subject of a recent detailed study [11]. As opposed to PAC, which stabilizes microtubules, colchicine has a destabilizing effect, which also results in the blockade of cell division [12]. Colchicine-mediated enhancement of VSV was attributed in part to a defect in IFN secretion by infected cells, thus preventing the cytokine-conferred antiviral protection [11].

The combination of PAC with OV treatment has been tested for vaccinia virus and herpes virus for other indications [13, 14]. This study focuses on the efficacy of MG1 for breast cancer treatment and investigates the co-treatment with PAC. Here, using three different murine breast cancer models, we demonstrate that MG1 can be enhanced by PAC both in vitro and in vivo and that the co-treatment improves efficacy better than either treatment on its own without impairing the safety profile of the virus.

## Methods

### Cell lines and culture

Vero kidney epithelial, 4 T1, EMT6 and EO771 murine mammary carcinoma and Hs578T, BT-549 and MDA-MB-231 human mammary carcinoma cell lines (American Type Culture Collection (Manassas, VA, USA)) were cultured in Dulbecco’s Modified Eagle’s Medium (DMEM) (Corning cellgro, Manassas, VA, USA) supplemented with 10 % fetal bovine serum (FBS) (Sigma life science, St-Louis, MO, USA) and maintained at 37 °C with 5 % CO_2_.

### Virus amplification and purification

MG1-green fluorescent protein (GFP) was purified as previously described [8]. Briefly, Vero cells were infected for 24 h at a multiplicity of infection (MOI) of 0.01. Supernatants were then filtered using a 0.2-um bottle top filter (Millipore, MA, USA) prior to 1.5-h centrifugation at 30100 g. The pellet was resuspended in Dulbecco’s phosphate-buffered saline (DPBS) (Corning cellgro, Manassas, VA, USA) and aliquots were stored at −80 °C.

### PAC treatment

PAC was purchased from Accord healthcare Inc. (Durham, NC). The cells were pre-treated at a concentration of 2 uM in culture media for 4 h prior to infection, unless specified otherwise. For in vivo experiments, animals were treated intraperitoneally (IP) with 2 mg/kg or 10 mg/kg of PAC as specified (see figure legends).

### Virus titration

Titers were obtained by plaque assay. Briefly, serial dilutions of the samples were transferred to monolayers of Vero cells. Following an incubation of 1 h, cells were overlaid with 0.5 % agarose/DMEM supplemented with 10 % FBS. Plaques were counted 24 h later. For in vivo experiments, tumors and organs were collected 48 h post treatment, homogenized in PBS using a tissue homogenizer, then serially diluted and virus quantified as described above.

### In vitro IFNβ treatment and quantification

Monolayers of tumor cells were treated with 250 U/mL of murine IFNβ (PBL interferon source, Piscataway, NJ, USA) 4 h prior to virus infection. The production of IFNβ by tumor cells was quantified using the ELISA mouse IFNβ kit (R&D systems, Minneapolis, MN, USA) following the manufacturer’s protocol. The samples were generated by pre-treating the cells with PAC as described above and infecting them for 24 h at an MOI of 0.1.

### Coomassie Blue staining/viability assay

At 72 h post infection, cells were fixed for 30 minutes using fixative solution (3:1 methanol-acetic acid). The fixative was then replaced by the Coomassie Blue staining solution (3:1 methanol-acetic acid, 0.1 % Commassie Blue dye) for 30 minutes. The plates were washed and dried overnight prior to scanning. For quantification, the Coomassie Blue staining was solubilized using 10 % SDS, and serial dilutions were performed and transferred to a 96-well plate for reading using a Fluoroscan plate reader at 450 nm.

### Microscopy

For nuclear staining, cells were cultured and treated on coverslips for 72 h. Cells were then washed with cold PBS and fixed using ice-cold methanol-acetone (1:1). Nuclei were stained using 4',6-diamidino-2-phenylindole (DAPI) included in the Prolong gold anti-fade (Molecular Probes) used to mount the coverslips onto slides. Live images of MG1-GFP infected cells were acquired using an EVOS Fl cell imaging system (ThermoFisher Scientific) microscope 24 h post infection.

### Flow cytometry

For quantification of virus infection, cells were processed as previously described [15]. Briefly, cells were harvested and fixed using IC fixation buffer (eBioscience) 24 h after PAC treatment and infection with MG1-GFP at an MOI of 0.01. Cells were then washed twice and resuspended in FACS buffer (3 % FBS, PBS) for analysis using a Cyan ADP 9 flow cytometer (Beckman Coulter, Mississauga, ON, Canada).

### In vivo experiments and tumor models

Balb/c mice were used (Charles River Laboratories) for the 4 T1 and EMT6 murine tumor models. For orthotopic implantation of the tumors, 2 × 10^5^ cells were injected into the second left mammary fat pad. For the EO771 tumor model, 1 × 10^6^ cells were implanted into the second left mammary fat pad of C57/Bl6 mice. For treatments, the virus and drug preparations were diluted to the appropriate concentration in a total volume of 100 uL of PBS and injected IP or intratumorally (IT) using insulin syringes (The Stevens Co, Montreal, QC, Canada). All experiments were performed in accordance with the University of Ottawa animal care and veterinary services guidelines.

### Histological analysis

Tumors were collected 48 h after treatment and fixed in 10 % buffered formalin phosphate (Fisher Scientific, Waltham, MA, USA) for 48 h. Paraffin-embedded sections were stained using hematoxylin and eosin or the specified antibodies. For antibody staining, the sections were rehydrated through graded alcohol and heat-mediated antigen retrieval was performed in citrate buffer (sodium citrate 10 mM, pH 6). Tissue sections were stained as described previously [16] using a rabbit anti-VSV (made in house) and rabbit anti-caspase-3 (Cell signalling technology) antibodies.

### TNBC ex-vivo samples

Patient-derived TNBC xenografts were grown into NOD/SCID mice as described previously [17, 18]. When the tumors reached 1500 mm^3^ in size they were collected and cores were generated as described previously [19]. The cores were treated ex-vivo with MG1 (10^3^ plaque-forming units (pfu)) and PAC and culture supernatant was collected 48 h later to titer the virus output.

### Tumor measurements and survival experiments

The length and width of the tumors were measured using digital calipers (Fowler). The formula (length × width^2^)/2 was used to calculate tumor volumes. The mice were sacrificed when they displayed respiratory distress, significant weight loss, ulceration, or discomfort, or when the tumor volume reached 1500 mm^3^ in size.

### Statistical analysis

Statistical analyses were performed using GraphPad Prism 6.0 software (see figure legends). Error bars represent standard error of the mean.

## Results

### PAC treatment enhances MG1 replication and killing in different breast cancer cell lines

**Fig. 1:**
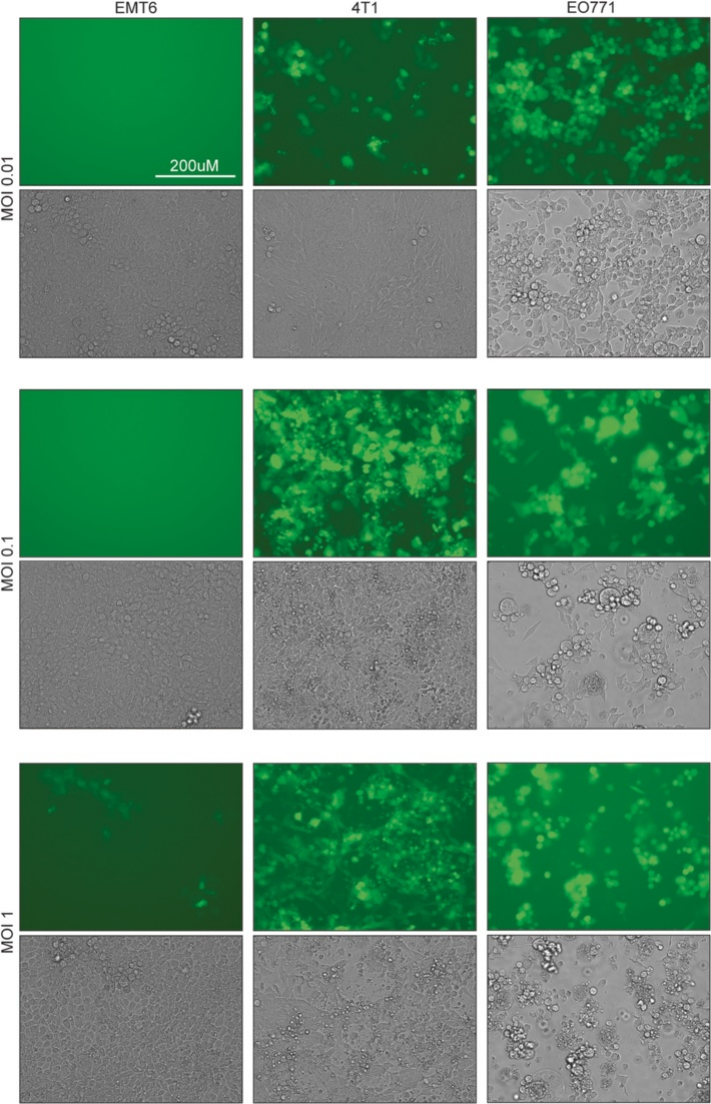
Murine breast cancer cell lines display different sensitivities to MG1. Microscopy images of EMT6, 4 T1 and EO771 tumor cells infected with various multiplicities of infection of MG1-green fluorescent protein for 24 h

In order to determine which concentration of drug and MOI of virus to use in our experiment, we first assessed the sensitivity of our different murine breast cancer cell lines to either treatment. Using a GFP-expressing version of MG1, we infected monolayers of cells at various MOIs and assessed transgene expression as a readout of infectivity. We also evaluated virus-mediated killing of the cells by looking at visible cytotoxicity. Our results demonstrate various sensitivities to the virus with the E0771 cells being completely infected at an MOI of 0.01 and all dead at an MOI of 10, and the EMT6 cells being the most resistant with only a few cells expressing GFP at an MOI of 10 (Fig. 1). The 4 T1 cells displayed an intermediate phenotype. We then investigated the sensitivity of the cell lines to PAC-induced killing. To do so, we incubated monolayers of cells with increasing concentrations of the drug for 48 h and performed a Coomassie Blue viability assay where decreased staining intensity reveals cytotoxicity. Similar to what we observed with the virus, the EO771 cells were the most sensitive to PAC and the EMT6 the most resistant cell line (Additional file 1: Figure S1a). The concentrations required to kill the various tumor cells ranged from 4 uM to 12 uM. To investigate the responsiveness of the cell lines to PAC-mediated inhibition of cellular division, we stained the nucleus of treated cells in order to detect enlarged, polynuclear cells. Interestingly, all three tumor cell lines were responsive to low, sub-lethal concentrations of PAC (0.5 uM) (Additional file 1: Figure S1b).

**Fig. 2:**
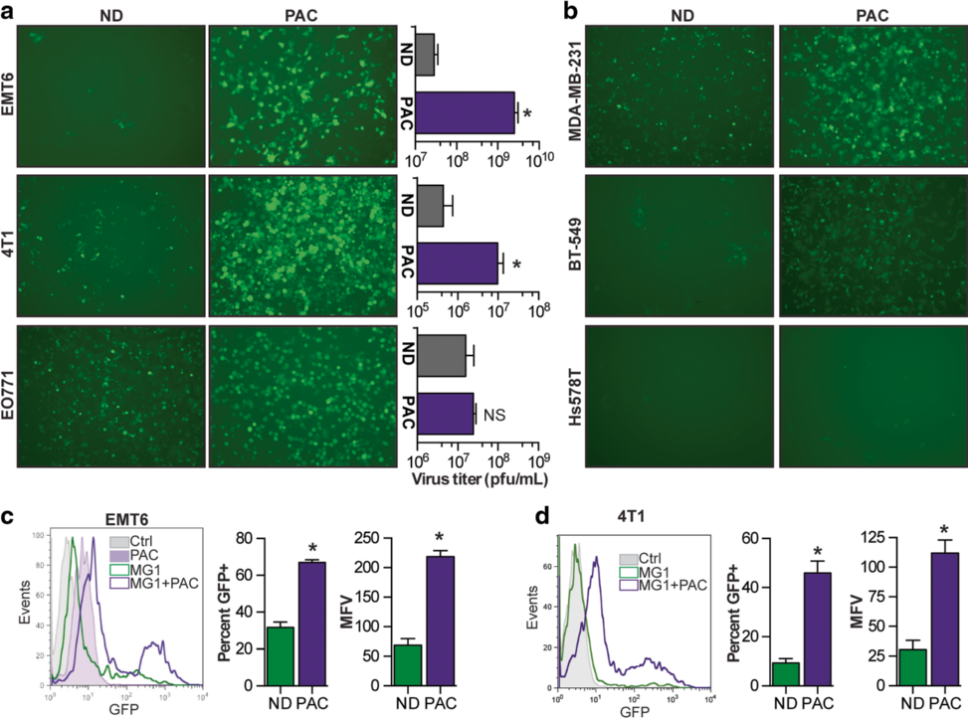
Paclitaxel (*PAC*) enhances MG1 in various human and mouse breast tumor cell lines. **a** Microscopy images of EMT6, 4 T1 and EO771 tumor cells infected with MG1-green fluorescent protein (*GFP*) after a 4-h pre-treatment with 2 uM PAC. Graphs *right* represent virus titers obtained 24 h post infection. *ND* no drug, *pfu* plaque-forming units. **b** Microscopy images of MDA-MB-231, BT-549 and Hs578T human tumor cells infected with MG1-GFP after a 4-h pre-treatment with 2 uM PAC. Flow cytometry histograms show the GFP expression of infected EMT6 (**c**) or (**d**) 4 T1 cells 24 h post infection with or without PAC pre-treatment. The right graphs show percentage of GFP+ cells and the mean fluorescence values (MFV). Samples were analyzed in triplicates. Statistical significance was tested using the unpaired two-tailed *t* test with Welch’s correction; *p < 0.05, **p < 0.01 ***p < 0.001. *Ctrl* control

To evaluate the effect of PAC on viral replication, we assessed the presence of GFP-positive cells following co-treatment with the drug in our murine breast cancer cell lines. We pre-treated the cells for 4 h with 2 uM of PAC, a concentration at which all cell lines displayed polynucleation, but none of them exhibited drug-mediated killing (Additional file 1: Figure S1). Our results indicate the presence of more MG1-infected EMT6 and 4 T1 cells for the co-treatment conditions (Fig. 2a, left panels). This enhancement was confirmed by quantification of the virus in the supernatants where 10-fold to 100-fold more virus was detected in the presence of the drug (Fig. 2a, right panels). For the E0771 cell line, no difference was observed in virus recovery and GFP expression, demonstrating that the drug did not enhance, but did not impair virus production either. We obtained similar results using three different human TNBC cell lines, whereby virus enhancement was observed in two cell lines (MDA-MB-231 and BT-549) but not in the third (Hs578T) (Fig. 2b). We used flow cytometry as a means to quantify the percentage of GFP-expressing cells and the mean fluorescence value (MFV) of infected cells in the presence or absence of the drug. Our results demonstrate that there were more GFP-positive cells when we pre-treated the 4 T1 and EMT6 cells with PAC, with percentages and average fluorescence twofold to threefold higher in the presence of the drug (Fig. 2c and d).

In a recent study we demonstrated that the viral sensitization mediated by colchicine, another drug affecting microtubules and preventing cell division was mediated by a blockade in the secretion of antiviral IFNs [11]. As many tumor cell lines are refractory to antiviral IFNs and would thus be refractory to enhancement involving this mechanism of action [7], we first assessed the sensitivity of our cell lines to the cytokine. Additional file 2: Figure S2a shows that pre-treating the cells with IFNβ efficiently protected all three cell lines against the virus. Consistent with this, less killing of the cells was observed with IFNβ pre-treatment (Additional file 3: Figure S3). To measure the IFNβ production in response to virus infection, we performed an ELISA on culture supernatants of infected cells. For all three cell lines, the cytokine was detected following infection. Interestingly, and consistent with our virus enhancement data (Fig. 2), our results show that in both EMT6 and 4 T1 cells, the production of IFNβ was impaired in the presence of PAC, while the levels produced by the E0771 cells were unaffected by the drug (Additional file 2: Figure S2b).

**Fig. 3:**
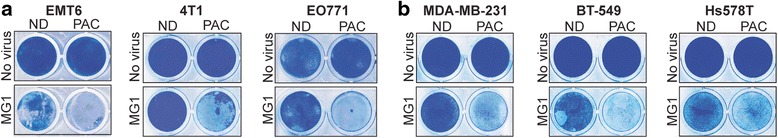
Paclitaxel (*PAC*) and MG1 synergistically kill breast cancer cell lines. Coomassie Blue staining of EMT6, 4 T1 and EO771 (**a**) and MDA-MB-231, BT-549 and Hs578T cells (**b**) infected or not with MG1-green fluorescent protein and co-treated with 2 uM PAC for 48 h. *ND* no drug

As the aim of both MG1 and PAC treatments is ultimately to kill tumor cells, we assessed cell death following co-treatment. We used a concentration of the drug where no cytotoxicity was observed following a 48-h incubation. For all three murine cell lines, we observed more cytotoxicity in the presence of both treatments with almost all the cells being dead, suggesting synergistic rather than cumulative killing (Fig. 3a). This decrease in viability was confirmed by quantification of the staining (Additional file 4: Figure S4). This synergistic killing was also confirmed using the MDA-MB-231, BT-549 and Hs578T human cell lines (Fig. 3b).

### The virus is enhanced by PAC treatment in vivo

**Fig. 4:**
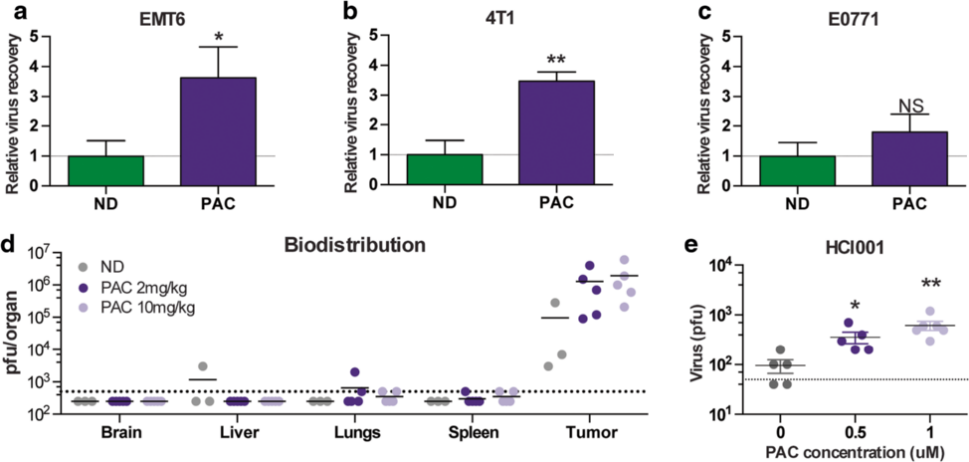
Paclitaxel (*PAC*) enhances MG1 replication in tumors. EMT6 (**a**), 4 T1 (**b**) or EO771 (**c**) tumor-bearing mice were treated intratumorally with 1 × 10^8^ plaque-forming units (*pfu*) of MG1-green fluorescent protein (GFP) and intraperitoneally with PAC (10 mg/kg for the EMT6 model and 2 mg/kg for the 4 T1 and EO771 models). Tumors were harvested 48 h later and the viral quantification was obtained by plaque assay. Three or more tumors per condition were analysed. **d** EMT6 tumor-bearing mice were treated as in **a** and various organs were collected 48 h post treatment. The virus was quantified by plaque assay. **e** Tumor cores from human triple-negative breast cancer xenografts were infected ex-vivo with MG1-GFP with or without PAC co-treatment. The viral outputs were quantified by plaque assay. Statistical significance was calculated using the unpaired two-tailed *t* test with Welch’s correction; *p < 0.05, **p < 0.01, ***p < 0.001. *ND* no drug

We then sought to confirm our in vitro findings in tumor-bearing animals. We implanted tumors orthotopically in order to recapitulate the natural microenvironment as much as possible. The virus was administered IT and quantified 48 h later by plaque assay. Our results demonstrate that threefold to fourfold more virus was detected in the EMT6 and 4 T1 tumors of the animals that also received PAC treatment compared to those that were treated with MG1 alone (Fig. 4a and b). For the E0771 tumor-bearing animals, we did not observe any difference in the amount of virus we recovered, consistent with the in vitro findings using this cell line (Figs. 2a and 4c). In order to assess if the increased viral replication would also result in increased replication in normal organs and impair the safety of the treatment, we performed a biodistribution experiment of MG1 48 h post injection in EMT6-tumor-bearing animals using the two drug concentrations used in this study. Our data demonstrate that, while both drug concentrations were able to increase viral replication in the tumors, no differences were observed in normal organs following co-treatment (Fig 4d).

To confirm the positive effect of PAC-treatment on MG1 infection of human tumors, we used a breast cancer patient xenograft model that was previously described to recapitulate the human disease [17, 18]. We infected tumor cores ex vivo in the presence or absence of PAC. Our results demonstrate that PAC efficiently enhanced viral replication in a TNBC patient-derived xenograft (Fig. 4e).

### PAC-MG1 combination therapy demonstrates greater tumor killing in vivo

**Fig. 5:**
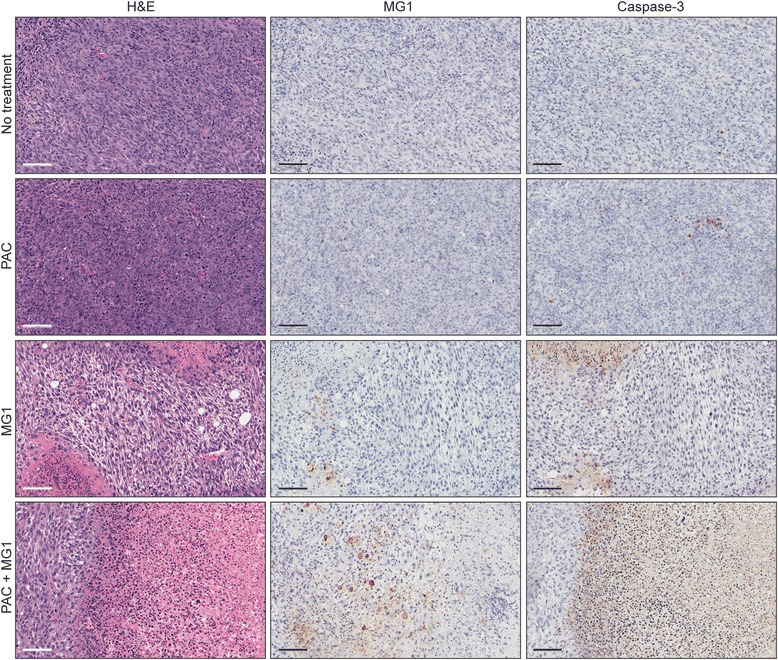
The co-treatment with paclitaxel (*PAC*) and MG1 increases tumor apoptosis. EMT6 tumor-bearing animals were treated intratumorally with MG1-green fluorescent protein (10^8^ plaque-forming units) and intraperitoneally with 10 mg/kg with PAC, and tumor samples were harvested 48 h post treatment. Sections were stained for the presence of virus and apoptotic cells (caspase-3)

Previous work by Lin et al*.* demonstrates that while both PAC and an oncolytic herpes simplex virus induce apoptosis, the combination of both was more effective in human anaplastic thyroid cancer cell lines [14]. To investigate if this was also the case using MG1 in our tumor models, we performed immunohistochemical analysis against the cleaved pro-apoptotic molecule caspase-3 on EMT6 tumors from mice that received the various treatments. First, the hematoxylin and eosin staining clearly demonstrated the presence of widespread necrotic regions for the MG1 as well as the MG1 and PAC co-treated tumors (Fig. 5, left panels). This was confirmed by caspase-3 staining, which was extensive in these tumors. (Fig. 5, right panels). These regions were larger and more abundant in the co-treated animals compared to those that received the MG1 treatment only. Also, consistent with the virus quantification shown in Fig. 4a, staining of the tumor sections with a virus-specific antibody demonstrated increased virus spread in the presence of PAC (Fig. 5, middle panels).

### The treatment with MG1 and PAC demonstrates greater efficiency in murine tumor models

**Fig. 6:**
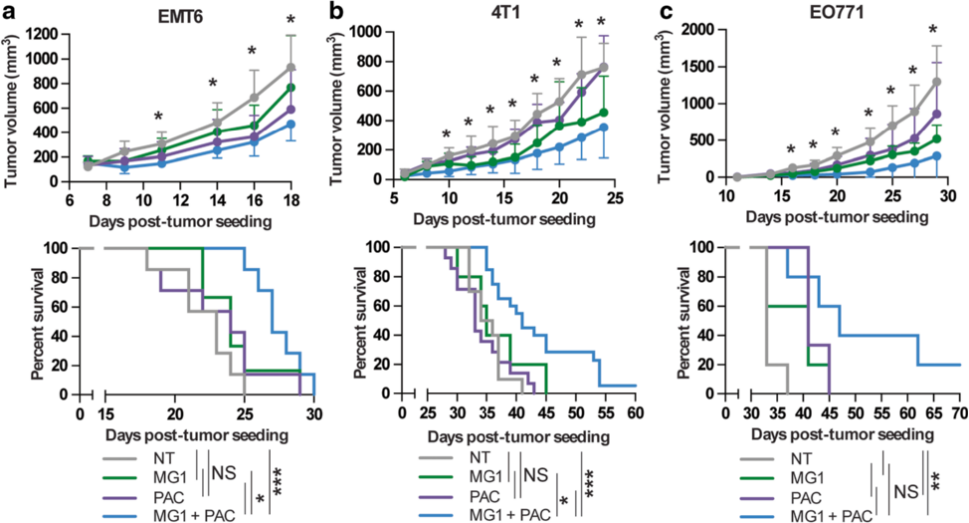
The combination of Paclitaxel (*PAC*) and MG1 improves efficacy in various murine breast cancer models. Volumes of EMT6 (**a**), 4 T1 (**b**) or EO771 (**c**) fat-pad tumors in mice treated or not (*NT*) with MG1-green fluorescent protein (1 × 10^8^ pfu) in combination or not with PAC (10 mg/kg for EMT6 and 2 mg/kg for 4 T1 and EO771). Mice were sacrificed when they reached the endpoint. *NS p* > 0.05, *p < 0.1, **p < 0.01, ***p < 0.001 (unpaired multiple two-tailed *t* test). Kaplan-Meier survival curves were generated from the same experiments (*bottom panels*). For survival experiments, *NS p* > 0.1, *p < 0.1, **p < 0.01, ***p < 0.001 (Mantel-Cox test)

Given the improved killing observed with PAC in all three cell lines in vitro, the increased virus recovery from tumors and the greater caspase-3-positive tumor regions observed in the presence of the co-treatment, we sought to determine if these phenomena would translate into slower tumor growth and an improvement in the survival of tumor-bearing animals. The mice were treated with MG1, PAC or both and tumors were measured over time. As expected, we observed that the growth was slower following treatment with the combination of the virus and the drug in all three tumor models (Fig. 6a, b and c, upper panels). Also, as observed in vitro, the E0771 model was the most sensitive to both treatments. Importantly, the enhanced control of tumor growth translated into a significant prolongation of survival in all three models and some animals were even cured for the 4 T1 and E0771 tumor models (Fig. 6a, b and c, bottom panels).

## Discussion

In this study, we demonstrated the compatibility of PAC, a standard-of-care chemotherapeutic agent for breast cancer, and MG1, an OV that is considered a promising and novel strategy for treating disseminated diseases like breast cancer. Our results not only show that treatments do not interfere with one another, but they can also perform even better when co-administered. Using three different syngeneic murine breast cancer models, we show a prolongation of survival for animals that received both treatments compared to either treatment alone (Fig. 6). These findings have potential implications for the future treatment of patients. Our data support clinical testing of the combination. Even if the patient’s cancer has become resistant to the drug, it might still effectively enhance MG1 and at the very least, should not impair the viral treatment. Second, because the beneficial effects we observed were achieved using sub-lethal concentrations of PAC and knowing the various side effects of the drug in patients with cancer, it is tempting to suggest that using a lower concentration of PAC in combination with the virus would be a suitable strategy.

Interestingly, we found that two out of the three murine tumor cell lines and the human tumor cell lines that we tested were sensitized to viral infection by PAC (Fig. 2). Indeed, while EMT6 and 4 T1 cells produced more virus when pre-treated with the drug, the E0771 cell line was not affected. Our model is that PAC pre-treatment would block the secretion of antiviral factors like IFNβ by infected cells (Additional file 2: Figure S2B), thereby increasing virus infection. In line with this idea, both EMT6 and 4 T1 cells in which we see sensitization to the virus, but not the E0771 cells, which are refractory to this effect, demonstrated impaired production of the antiviral cytokine (Additional file 2: Figure S2B). Importantly, our results also show that while ex-vivo infection and treatment of patient breast cancer xenografts can inform us on the potential enhancement of the virus by the drug in specific patient samples (Fig. 4e), the lack of enhancement would not necessarily imply that both treatments would not be compatible. Indeed, increased production of virus is desirable, but the killing of the target tumor cells is what ultimately matters. Our data show that although the E0771 cell line does not demonstrate sensitization to the virus in the presence of PAC, complete killing of the cells was still observed at concentrations of PAC and MG1 that did not affect cell viability alone. Notably, the E0771 tumor model was the one in which we observed the highest percentage of cures with the treatment combination (Fig. 6c). This is most likely due to the greater sensitivity of the E0771 tumors to both single treatments alone compared to the two other tumor models (Fig. 6). Remarkably, while the EMT6 and 4 T1 tumors are slightly smaller compared to the control animals when treated with either the virus or the drug, the combination of both treatments was the only condition that was significantly different in terms of the ability to control tumor growth and prolong survival.

An interesting idea to explain the improved killing observed in vitro in the E0771 cell line would be that virus-induced factors may promote PAC killing. Indeed, it has been reported by Thorne and colleagues that oncolytic vaccinia virus induces the secretion of factors, including type I IFNs, which sensitize tumor cells to taxol [13]. Also, a similar mechanism was observed for the colchicine and VSV co-treatment [11]. This scenario would provide a mechanism by which even tumors that are refractory to the PAC-mediated sensitization to MG1 could still benefit from the combination. Of note, infection with vaccinia virus produces various anti-inflammatory factors [20], including B18R which inhibits the activity of IFNs and could potentially minimize this effect, while MG1 and VSV do not encode these inhibitors, suggesting that the virus-mediated sensitization of tumor cells to PAC-mediated killing could be even greater [7, 8].

Another interesting application of our combination strategy would be to potentiate the production of virally-encoded transgenes. The engineering of oncolytic viruses has been shown to be a successful strategy for tumor-targeted gene delivery and improvement of treatment efficacy. Indeed, rhabdoviruses encoding antiviral suppressors to increase viral replication [21], suicide genes to improve killing [22] or immune-stimulating cytokines to induce a greater anti-tumor immune response [23] have all been shown to control tumor growth more efficiently compared to the parental virus. Given that, along with the increased virus production, we also observed more GFP-positive cells and higher MFV (Fig 2b and c) and thus, greater transgene production, the co-treatment could potentially be even more beneficial using viruses that encode transgenes that mediate greater control of the tumors.

## Conclusions

With the urgent need for novel strategies for breast cancer treatment, especially in patients with TNBC, which are refractory to the limited available treatment options, our work provides a rational alternative to improve outcomes. Our data demonstrate that the combination of PAC and MG1 is effective at controlling tumor progression. Because PAC is a standard of care for breast cancer treatment and MG1 is undergoing clinical testing, we believe the findings included in this study are of great importance and that the translation of this work to the clinic could be rapid.

## Abbreviations

DAPI, 4',6-diamidino-2-phenylindole; DMEM, Dulbecco’s modified Eagle’s medium; ELISA, enzyme-linked immunosorbent assay; FBS, fetal bovine serum; GFP, green fluorescent protein; IFN, interferon; IP, intrperitoneally; IT, intratumorally; MFV, mean fluorescence value; MOI, multiplicity of infection; OV, oncolytic virus; PAC, paclitaxel; PBS, phosphate-buffered saline; pfu, plaque-forming units;TNBC, triple-negative breast cancer; VSe, virus sensitizers; VSV, vesicular stomatitis virus

## Additional files

Additional file 1: Figure S1. Murine breast cancer cell lines display different sensitivities to PAC. **A** EMT6, 4 T1 and EO771 cells were treated with increasing concentrations of PAC. Coomassie Blue staining was used to assess the toxic dose of the drug in the different cell lines after 72 h. **B** Fluorescent microscopy pictures of DAPI-stained cells with or without treatment with 0.5uM PAC for 72 h. (TIF 14849 kb) Additional file 2: Figure S2. PAC blocks IFNβ production by infected tumor cells. **A** Microscopy pictures of EMT6, 4 T1 and EO771 tumor cells pre-treated or not for 4 h with recombinant IFNβ. **B** The IFNβ released from MG1-infected cells in the presence or absence of PAC was quantified by ELISA. Samples were analysed in triplicates and statistical significance was calculated using the unpaired two-tailed *t* test with Welch’s correction; **p* < 0.05, ***p* < 0.01, ****p* < 0.001. (TIF 6796 kb) Additional file 3: Figure S3. IFNβ pre-treatment protects EMT6, 4 T1 and EO771 cells from virus-mediated killing. Coomassie Blue staining of the various breast cancer cell lines 48 h post infection with MG1-GFP with or without pre-treatment with recombinant murine IFNβ. (TIF 2149 kb) Additional file 4: Figure S4. PAC and MG1 synergistically kill breast cancer cell lines. Quantification of the signal obtained for triplicates or quadruplicates Coomassie Blue staining of EMT6, 4 T1 or EO771 cells infected or not with MG1-GFP and co-treated with 2 uM PAC for 48 h from Fig. 3. Statistical significance was calculated using the unpaired two-tailed *t* test with Welch’s correction; **p* < 0.05, ***p* < 0.01, ****p* < 0.001. (TIF 2942 kb)
