# Supplementary figures and images for: Combination of Paclitaxel and MG1 oncolytic virus as a successful strategy for breast cancer treatment
Source: Breast Cancer Res. 2016 Aug 8;18:83. doi: 10.1186/s13058-016-0744-y (PMC4977613; doi:10.1186/s13058-016-0744-y)

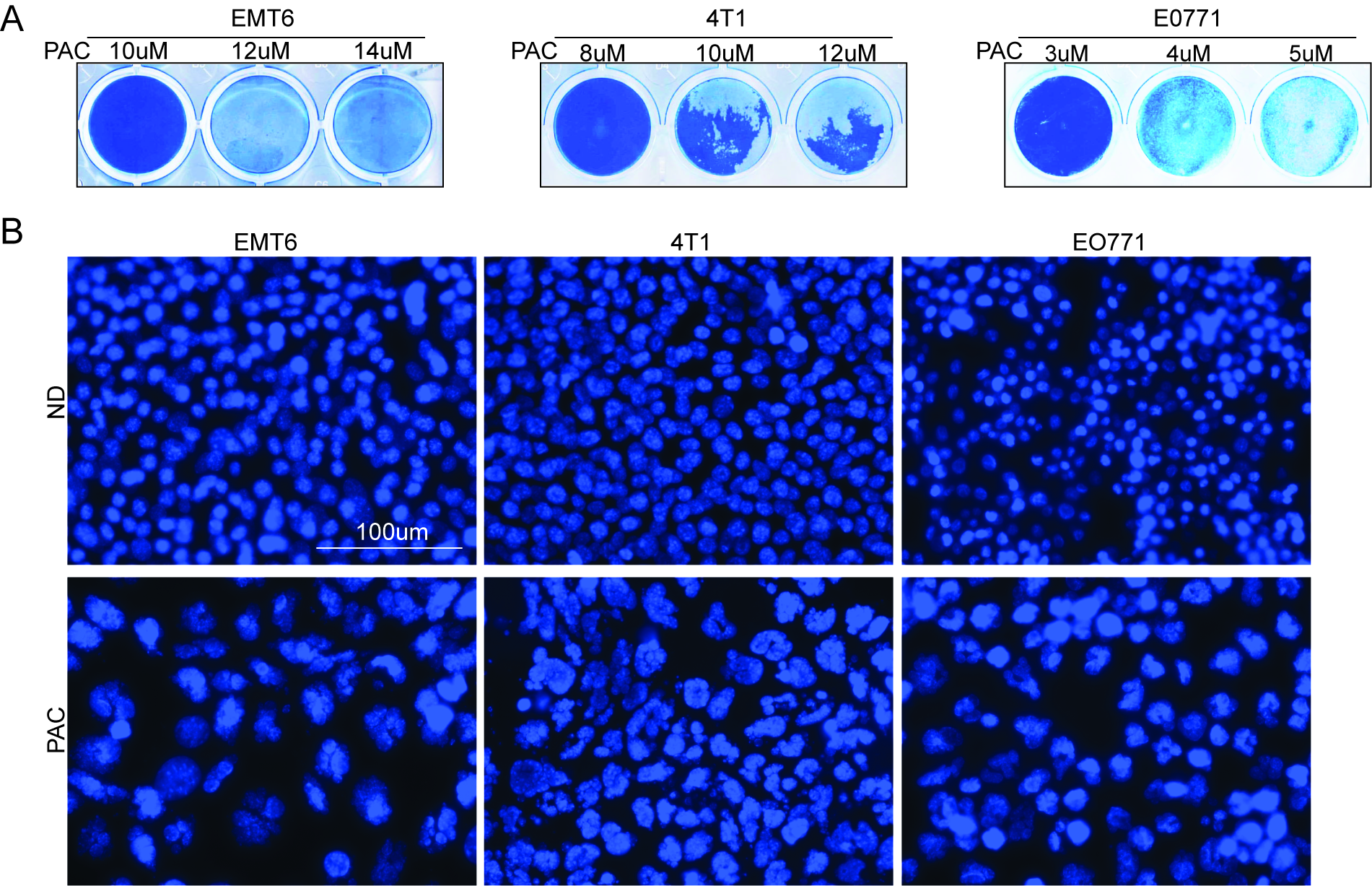

Supplement: Additional file 1: Figure S1. — Murine breast cancer cell lines display different sensitivities to PAC. A EMT6, 4 T1 and EO771 cells were treated with increasing concentrations of PAC. Coomassie Blue staining was used to assess the toxic dose of the drug in the different cell lines after 72 h. B Fluorescent microscopy pictures of DAPI-stained cells with or without treatment with 0.5uM PAC for 72 h. (TIF 14849 kb) [file 13058_2016_744_MOESM1_ESM.tif]

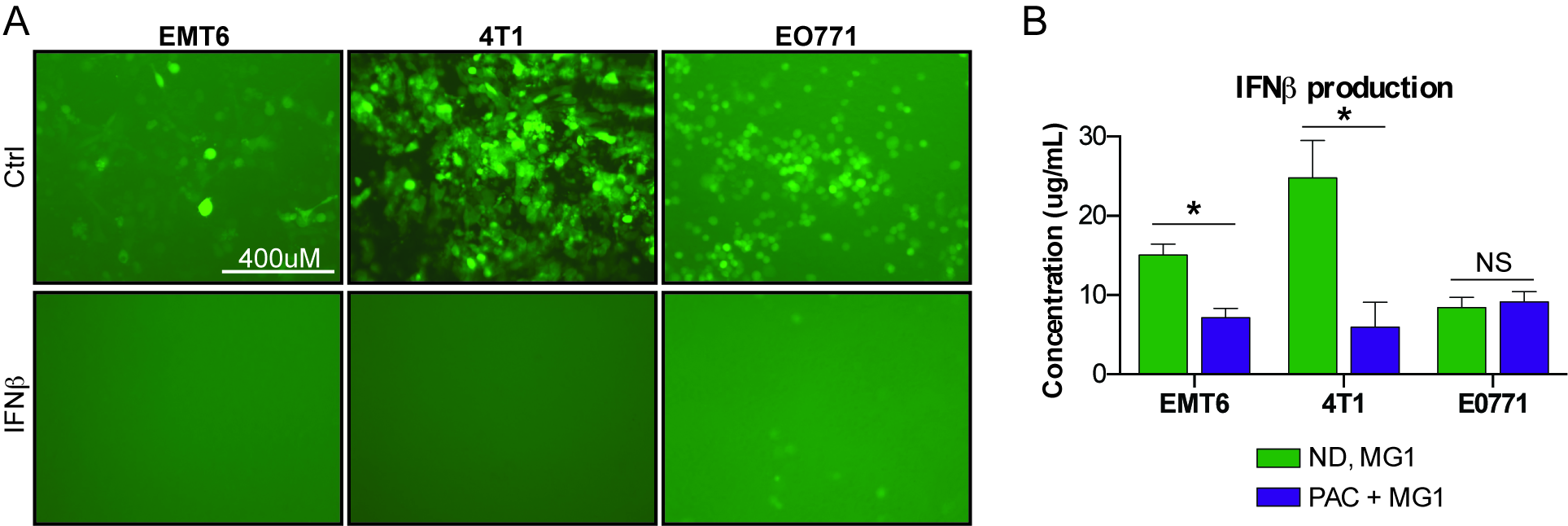

Supplement: Additional file 2: Figure S2. — PAC blocks IFNβ production by infected tumor cells. A Microscopy pictures of EMT6, 4 T1 and EO771 tumor cells pre-treated or not for 4 h with recombinant IFNβ. B The IFNβ released from MG1-infected cells in the presence or absence of PAC was quantified by ELISA. Samples were analysed in triplicates and statistical significance was calculated using the unpaired two-tailed t test with Welch’s correction; *p < 0.05, **p < 0.01, ***p < 0.001. (TIF 6796 kb) [file 13058_2016_744_MOESM2_ESM.tif]

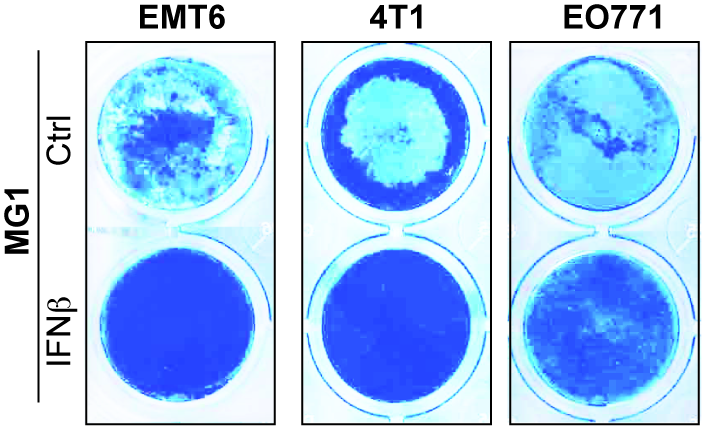

Supplement: Additional file 3: Figure S3. — IFNβ pre-treatment protects EMT6, 4 T1 and EO771 cells from virus-mediated killing. Coomassie Blue staining of the various breast cancer cell lines 48 h post infection with MG1-GFP with or without pre-treatment with recombinant murine IFNβ. (TIF 2149 kb) [file 13058_2016_744_MOESM3_ESM.tif]

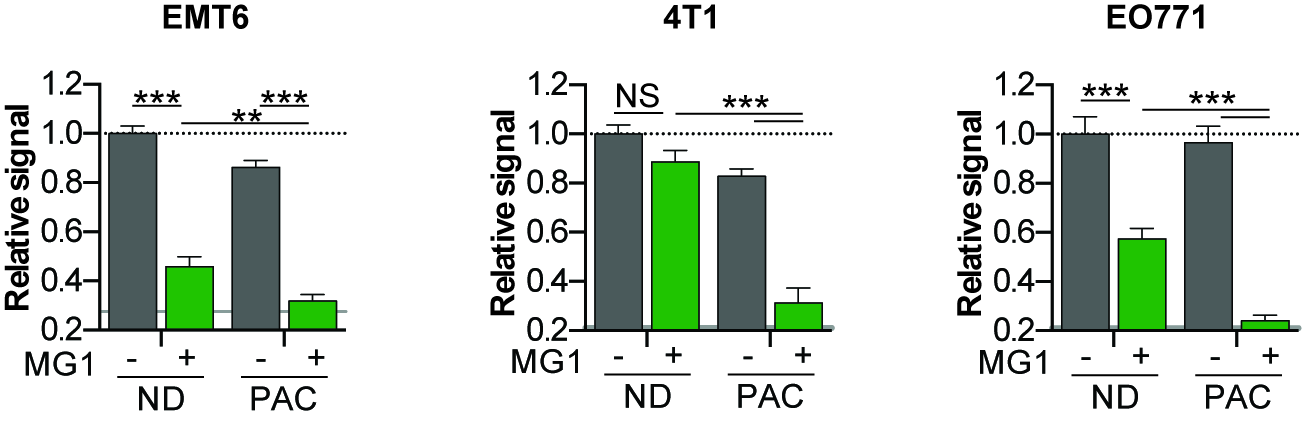

Supplement: Additional file 4: Figure S4. — PAC and MG1 synergistically kill breast cancer cell lines. Quantification of the signal obtained for triplicates or quadruplicates Coomassie Blue staining of EMT6, 4 T1 or EO771 cells infected or not with MG1-GFP and co-treated with 2 uM PAC for 48 h from Fig. 3. Statistical significance was calculated using the unpaired two-tailed t test with Welch’s correction; *p < 0.05, **p < 0.01, ***p < 0.001. (TIF 2942 kb) [file 13058_2016_744_MOESM4_ESM.tif]
